# Detecting COVID-19 patients via MLES-Net deep learning models from X-Ray images

**DOI:** 10.1186/s12880-022-00861-y

**Published:** 2022-07-30

**Authors:** Wei Wang, Yongbin Jiang, Xin Wang, Peng Zhang, Ji Li

**Affiliations:** 1 School of Computer and Communication Engineering, Changsha University of Science and Technology, Changsha, 410114 China; 2 School of Electronics and Communications Engineering, Sun Yat-Sen University, Shenzhen, 518107 China

**Keywords:** COVID-19, Deep learning, MLES-Net, Convolutional neural network (CNN), Chest X-Ray images

## Abstract

**Background:**

Corona Virus Disease 2019 (COVID-19) first appeared in December 2019, and spread rapidly around the world. COVID-19 is a pneumonia caused by novel coronavirus infection in 2019. COVID-19 is highly infectious and transmissible. By 7 May 2021, the total number of cumulative number of deaths is 3,259,033. In order to diagnose the infected person in time to prevent the spread of the virus, the diagnosis method for COVID-19 is extremely important. To solve the above problems, this paper introduces a Multi-Level Enhanced Sensation module (MLES), and proposes a new convolutional neural network model, MLES-Net, based on this module.

**Methods:**

Attention has the ability to automatically focus on the key points in various information, and Attention can realize parallelism, which can replace some recurrent neural networks to a certain extent and improve the efficiency of the model. We used the correlation between global and local features to generate the attention mask. First, the feature map was divided into multiple groups, and the initial attention mask was obtained by the dot product of each feature group and the feature after the global pooling. Then the attention masks were normalized. At the same time, there were two scaling and translating parameters in each group so that the normalize operation could be restored. Then, the final attention mask was obtained through the sigmoid function, and the feature of each location in the original feature group was scaled. Meanwhile, we use different classifiers on the network models with different network layers.

**Results:**

The network uses three classifiers, FC module (fully connected layer), GAP module (global average pooling layer) and GAPFC module (global average pooling layer and fully connected layer), to improve recognition efficiency. GAPFC as a classifier can obtain the best comprehensive effect by comparing the number of parameters, the amount of calculation and the detection accuracy. The experimental results show that the MLES-Net56-GAPFC achieves the best overall accuracy rate (95.27%) and the best recognition rate for COVID-19 category (100%).

**Conclusions:**

MLES-Net56-GAPFC has good classification ability for the characteristics of high similarity between categories of COVID-19 X-Ray images and low intra-category variability. Considering the factors such as accuracy rate, number of network model parameters and calculation amount, we believe that the MLES-Net56-GAPFC network model has better practicability.

## Background

Corona Virus Disease 2019 (COVID-19) first appeared in December 2019, and spread rapidly around the world. COVID-19 is a pneumonia caused by novel coronavirus infection in 2019. COVID-19 is highly infectious and transmissible. Early in the epidemic, countries do not develop effective prevention measures in a timely manner. This has led to a surge in infections and deaths globally, and put pressure on health workers to diagnose and treat COVID-19. According to official reports from the WORLD Health Organization and countries (regions), by 7 May 2021, the total number of cumulative confirmed cases in the world is 156,400,486, the cumulative number of cured cases is 126,943,387, and the cumulative number of deaths is 3,259,033, with a mortality rate of 2.084%.

COVID-19 is an acute infectious pneumonia in which the X-Ray image of a virus-infected person is characterized by small patches or interstitial changes, with a shallow, fuzzy edge density and no consolidation characteristics [1]. Currently, vaccines and drugs against COVID-19 have not been widely used. Therefore, in order to diagnose the infected person in time to prevent the spread of the virus, the diagnosis method for COVID-19 is extremely important. In most areas, Reverse Transcription-Polymerase Chain Reaction (RT-PCR) is the main method to detect COVID-19. RT-PCR mainly collects respiratory specimens for detection, such as oropharyngeal swabs or nasopharyngeal samples. However, due to its disadvantages such as low sensitivity in the early stage and long waiting time for tests, patients may spread COVID-19 infection further while staying in the hospital for observation. In addition, due to the high environmental conditions required for testing and the lack of testing facilities, it cannot be promoted in some areas, which also leads to some limitations in the diagnosis of COVID-19. Therefore, finding an efficient method of screening infection is the key to reducing the limitations of diagnosing COVID-19.

Convolutional neural networks (CNN) have developed rapidly in the field of computer vision. Wang et al. [2] analyzed the application and development of CNN. LeNet [3] proposed by Lecun in 1998 was created to solve the handwritten digit recognition task. In 2012, Hinton et al. [4] proposed AlexNet. Due to insufficient computing power of the GPU and insufficient memory capacity at that time, the authors innovatively proposed a parallel network structure. The most important thing is that the AlexNet network introduced the ReLU nonlinear activation function, the dropout layer, and the local standardization layer. In the 2012 ImageNet image recognition competition, by using AlexNet, the error rate was increased from 25 to 15%.

VGGNet [5], designed by DeepMind and the Computer Vision Group of University of Oxford, is a deep convolutional neural network, which won the first place in the positioning project and the second place in the classification project in the ILSVRC-2014.

In 2015, Kaiming et al. [6] found that after adding identity to the neural network, even if the number of network layers reached 1200, the error rate was only 3.6%. After introducing Group convolution [4], Attention [7], Dense connection [8], Channel-Wise Attention [9], the error rate of network dropped to 2.2%. Later, Google researchers designed a new structure, inception module, to increase network depth and width while reducing parameters. After proposing inception V1 [10], inception V2 [11], V3 [12], and V4 [13] were put forward one after another. In this network structure, the amount of parameters and calculations are obviously reduced, but the nonlinear expression ability of the network becomes stronger. Kaiming’s team [14] proposed a residual structure to prevent gradient dispersion. Although the core block of ResNet is simple, the skip connect can almost perfectly solve the problem of network gradient disappearance. At the same time, the network training speed and feature extraction ability are improved by adding BN layers and increasing the network depth. Based on MobileNet [15], Wang et al. [8] introduced dense blocks to DenseNet and constructed a Dense-MobileNet [16], which further reduced the amount of network calculations and parameters, and achieved higher accuracy rate.

In recent years, artificial intelligence (AI) technology has gradually entered all areas of life due to its outstanding performance [17]. In particular, the deep learning method based on data classification, image segmentation and target detection has achieved excellent results in the application of medical field. For example, for making better use of the feature information in the image, and improving the network convergence speed, DRD-Net [18] uses the residual-dense structure for local feature fusion, and finally carries out global residual fusion reconstruction. Dense-MobileNet [16] reduces the amount of network parameters and computational cost by introducing dense blocks. Wang et al. [19] proposed an improved deep learning method for detecting colon polyp images, which achieved good results. Carrer et al. [20] proposed a pleural line detector to accurately retrieve the pleural line features in the image. Studies have proved that the AI-based computer-aided diagnosis system can provide rapid detection and diagnosis results, and help to screen and diagnose suspected COVID-19 cases [21]. Since COVID-19 is accompanied by common complications such as lung infection or pneumonia, CT or X-Ray can be used for diagnosis based on this point, which greatly accelerates the speed of detection and screening of COVID-19 cases. Although CT images can provide more details about case information, they cannot be widely promoted in most poor and underdeveloped areas because of the high price. Imaging radiology technology (X-Ray and CT) [22, 23], as an important supplement to the detection of the specific sequence of the new coronavirus (COVID-19). Ullah et al. [24] reviewed the various types of scalable Telehealth services used to support patients infected by COVID-19 and other diseases during this pandemic. Islam et al. [25] discussed the different existing wearable monitoring devices (respiration rate, heart rate, temperature, and oxygen saturation) and respiratory support systems (ventilators, CPAP devices, and oxygen therapy) which were frequently used to assist the coronavirus affected people. Islam et al. [26] overviewed the existing technologies which were frequently used to support the infected patients for respiration. They described the most recent developed breathing aid devices such as oxygen therapy devices, ventilator, and CPAP throughout the review. Rahman et al. [27] proposed a system that restricts the growth of COVID-19 by finding out people who were not wearing any facial mask in a smart city network where all the public places were monitored with Closed-Circuit Television (CCTV) cameras. Islam et al. [28] overviewed the recently developed systems based on deep learning techniques using different medical imaging modalities like Computer Tomography (CT) and X-Ray. Muhammad et al. [29] proposed that data mining models were developed for the prediction of COVID-19 infected patients’ recovery using epidemiological dataset of COVID-19 patients of South Korea. Asraf et al. [30] discussed the overall applications of deep learning on multiple dimensions to control novel coronavirus (COVID-19). According to the characteristics of chest X-Ray image, Wang et al. [31] designed the channel feature weight extraction (CFWE) module, and proposed a new convolutional neural network, CFW-Net to detect COVID-19 images.

AI is also playing an increasingly important role in medical diagnosis. Dark-COVID-Net [32] and a new network based on CovXNet [33] used neural network models to assist doctors in analyzing the infected area in X-Ray images. Rajaraman et al. [34] and Elaziz et al. [35] used machine learning method to detect the infection images in X-Ray images on the basis of feature extraction.

## Methods

### Attention mechanism

In recent years, deep learning has developed rapidly, and neural networks (NN) have achieved very good results in image recognition. Meanwhile, attention mechanism has been frequently appeared in some papers or blog posts so as to be a popular concept in NN. In this paper, “Recurrent Models of Visual Attention” [36] from the Google Mind team, which uses the Attention mechanism on the RNN model, is used in our new deep learning net to detect COVID-19 images. Bahdanau et al. [23] used a mechanism similar to attention to perform translation and alignment simultaneously in machine translation tasks. There are many different models of human visual attention, but they all basically boil down to giving more important attention to the target areas that need to be focused (the focus of attention), while giving lower attention to other areas, and then adjusting the focus over time. The attention mechanism in computer vision is essentially similar to the selective visual attention mechanism of human beings. Its core goal is to select the information that is more critical to the current task from a large amount of information.

### Model principle

In recent years, most of the researches on the combination of deep learning and visual attention mechanism have focused on the formation of attention mechanism by using masks. The principle of mask is to identify the key features in the image data through another layer of new weight. Through learning and training, the deep neural network learns the areas that need attention in each new image to form attention. The attention mechanism evolved into two different types, one called soft attention and the other called hard attention. The key point of soft attention is to pay more attention to space or channel. Soft attention is deterministic attention, which can be generated directly through the network after learning is completed. The most important thing is that soft attention is differentiable. Differentiable attention can be calculated by neural network gradient, and its weight can be learned by forward propagation and backward feedback. In the early stage of COVID-19, chest X-Ray may show small patchy shadows or interstitial changes, with shallow and fuzzy edge density and no consolidation characteristics. In addition, the chest X-Ray image has high similarity between categories and low intra-category variability, which will lead to model deviation and overfitting, and reduce the performance and generalization. The experimental results proved that the combination of soft attention mechanism and deep learning can achieve good effect in X-Ray image recognition of COVID-19. So, this paper introduces a Multi-Level Enhanced Sensation (MLES) module, and proposes a new convolutional neural network model, MLES-Net, based on this module. Although the attention mask is used at each level, it generates almost no extra computation by using the correlation between global and local features to generate the "Attention Mask". Compared with other methods, MLES-Net has higher recognition accuracy and stronger generalization ability.

## Dataset

### The dataset source

The experiments use two open source data sets. The chest X-Ray dataset of COVID-19 comes from GitHub (https://github.com/ieee8023/covid-chestxray-dataset), which consists of X-Ray images of different patients infected with COVID-19 and other pneumonia. It contains a total of 760 images, and we selected 412 X-Ray images of COVID-19 as positive patients. The second data set from Kaggle chest X-Ray images (https://www.kaggle.com/paultimothymooney/chest-xray-pneumonia) contains 5863 X-Ray images, which is divided into two categories: normal images and pneumonia images.

### Data classification

**Fig. 1:**
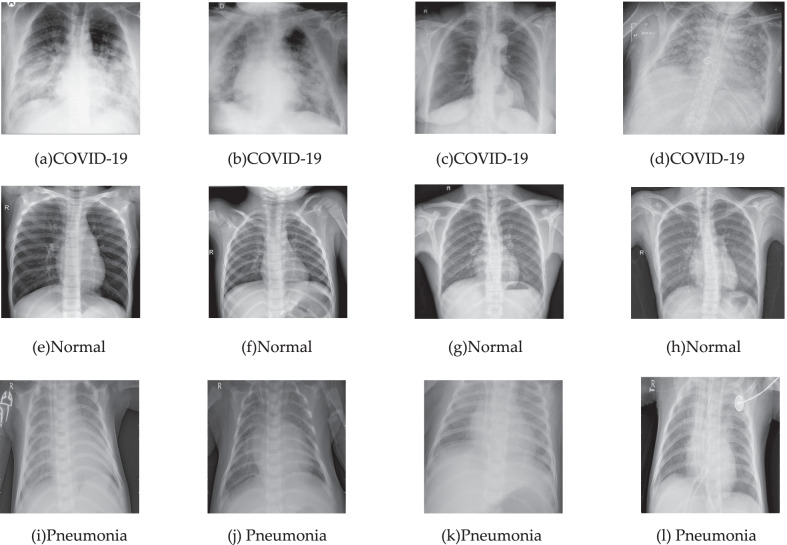
Chest X-Ray images

From this data set, we selected 4265 pneumonia images and 1575 normal images. Our training set contains 5526 X-Ray images, including 310 COVID-19 patient images, 1341 normal images, and 3875 normal pneumonia images. The test set contains 726 X-Ray images, including 102 COVID-19 patient images, 234 normal images, and 390 ordinary pneumonia images. The twelve sample images from the dataset that we have established are shown in Fig. 1a, b, c and d belong to the X-Ray images of COVID-19 patients. (e), (f), (g) and (h) belong to the X-Ray images of normal people. (i), (j), (k) and (l) belong to the X-Ray images of pneumonia patients without COVID-19.

From Fig. 1, it can be seen that the similarity between X-Ray image categories is high and the intra-category variability is low, which increases the recognition difficulty [22].

## MLES module

### MLES module

In order to provide doctors with possible computer-aided diagnosis, we designed a new deep learning network to detect COVID-19 infections from X-Ray images. Early chest X-Ray images of COVID-19 infected persons may show small patches or interstitial changes with shallow, blurry edge densities. Moreover, X-Ray images of the infected chest showed high inter-category similarity and low intra-category variability. When CNN is used for detection of infected persons, these two characteristics will lead to model deviation and over-fitting, which will lead to reduced performance and generalization.

**Fig. 2:**
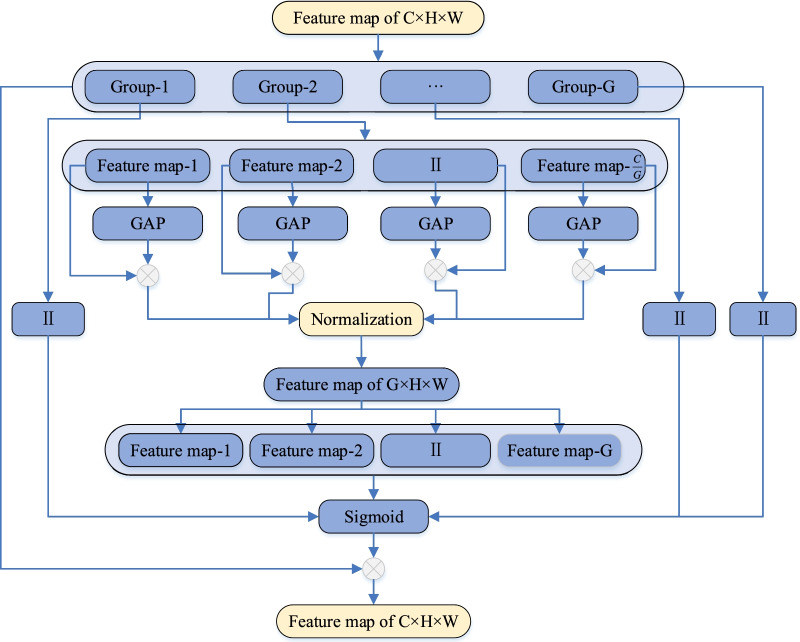
The structure of MLES module

**Fig. 3:**
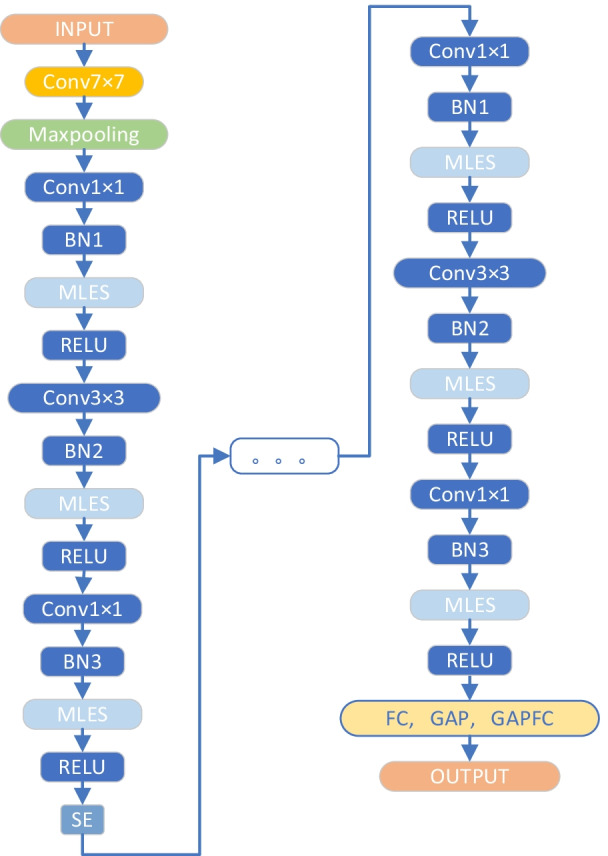
The main structure of MLES-Net

Aiming at the above problems, this paper designs a Multi-Level Enhanced Sensation Module (MLES module), and this paper proposes a new MLES-Net convolutional neural network model based on this module, which improves the X-Ray image recognition ability for COVID-19. The structure of MLES module is shown in Fig. 2. The main structure of MLES-Net is shown in Fig. 3. Since the middle part is the same as the display part, and only the network layers are different, the whole structure is not shown. Among them, “Agv_pool” means global average pooling layer, F(x) means feature average function, “Normalization” means regular normalization, “sigmoid” means activation function, and “⨂” means dot product.

### MLES architecture

We divide a multi-channel into multiple channel groups along its channel dimension. The network will carry out feature extraction in each channel group, including global and local feature. Since the activation area is determined by the global and local dependencies, each channel group has a global receptive field to a certain extent, and the output dimension is the same as the input characteristic channels. As many groups adopt parallel stacked topological structures [37], which is similar to model aggregation, the effect is better. At the same time, it also means that the global distribution of response in the characteristic channel group enables the layer near the input to obtain a good global receptive field.

If the input feature map size is H × W, the number of channels is C, and the feature map is divided into multiple channel groups G, so there are $\frac{{\text{C}}}{{\text{G}}}$ feature maps in each group. Assuming that under ideal circumstances, we can obtain a strong feature response in a certain area of the input picture, but due to the existence of similar patterns, it is usually difficult for the network to obtain a well-distributed feature response. Therefore, in the module construction, first, we take the global average pooling (GAP) operation on $\frac{{\text{C}}}{{\text{G}}}$ feature maps of each group, to approximate the implication of the group represent. By multiplying with the previous feature maps, dot product measures the similarity between the global semantic feature and local feature to some extent, and we get $\frac{{\text{C}}}{{\text{G}}}$ feature maps. In order to avoid the influence of the offset of coefficients between different samples, we use normalization. After the feature maps of each channel group being normalized and fused, we get the feature maps of G × H × W. Later, we repeat the above steps. After obtaining G feature maps of H × W in G channel groups, the sigmoid function is used to obtain the enhanced feature maps. Finally, we multiply the activated feature maps with the $\frac{{\text{C}}}{{\text{G}}}$ feature maps of each group at the beginning, to obtain the activated feature groups in different regions.

Attention mechanism both has the ability to automatically focus to the key points in a variety of information [38], so it can be used to improve the efficiency of the module. Attention enhancement mechanism is used in the module, and the channel is divided into several sub-channels with different semantics. Due to the lack of important generation features in sub-channels, the learning ability and generalization ability of network will be seriously weakened. For enhancing the feature extraction ability of the network, this module extracts features in both global and local areas to get a well-distributed feature map.

The global characteristic response is:

1 $$g = F_{{g_{P} }} \left( X \right) = \frac{1}{m}\mathop \sum \limits_{i = 1}^{m} x_{i}$$

The local characteristic response is:

2 $$c_{i} = g \times x_{i}$$

The Sigmoid is:

3 $$\hat{x}_{i} = x_{i} \times \sigma \left( {a_{i} } \right)$$

## MLES-Nets

Based on the MLES module, we propose the Multi-level enhance sensation networks (MLES-Net), as shown in Table 1.

**Table 1:** MLES-Net configuration

MLES-Net40		MLES-Net56		MLES-Net107	
		Conv7-64, stride:2 3 × 3Maxpool, stride:2			
Input-64 Conv3 × 3 BN1 MLES ReLU Conv3 × 3 BN2 MLES ReLU Output-64	× 3	Input-64 Conv1 × 1 BN1 MLES ReLU Conv3 × 3 BN2 MLES ReLU Conv1 × 1 BN3 MLES ReLU Output-256	× 3	Input-64 Conv1 × 1 BN1 MLES ReLU Conv3 × 3 BN2 MLES ReLU Conv1 × 1 BN3 MLES ReLU Output-256	× 3
		SE Module			
Input-128 Conv3 × 3 BN1 MLES ReLU Conv3 × 3 BN2 MLES ReLU Output-128	× 4	Input-128 Conv1 × 1 BN1 MLES ReLU Conv3 × 3 BN2 MLES ReLU Conv1 × 1 BN3 MLES ReLU Output-512	× 4	Input-128 Conv1 × 1 BN1 MLES ReLU Conv3 × 3 BN2 MLES ReLU Conv1 × 1 BN3 MLES ReLU Output-512	× 4
		SE module			
Input-256 Conv3 × 3 BN1 MLES ReLU Conv3 × 3 BN2 MLES ReLU Output-256	× 6	Input-256 Conv1 × 1 BN1 MLES ReLU Conv3 × 3 BN2 MLES ReLU Conv1 × 1 BN3 MLES ReLU Output-1024	× 6	Input-256 Conv1 × 1 BN1 MLES ReLU Conv3 × 3 BN2 MLES ReLU Conv1 × 1 BN3 MLES ReLU Output-1024	× 23
		SE module			
Input-512 Conv3 × 3 BN1 MLES ReLU Conv3 × 3 BN2 MLES ReLU Output-512	× 3	Input-512 Conv1 × 1 BN1 MLES ReLU Conv3 × 3 BN2 MLES ReLU Conv1 × 1 BN3 MLES ReLU Output-2048	× 3	Input-512 Conv1 × 1 BN1 MLES ReLU Conv3 × 3 BN2 MLES ReLU Conv1 × 1 BN3 MLES ReLU Output-2048	× 3
		FC, GAP, GAPFC			
		OUTPUT			

Compared with AlexNet, VGGNets and other networks which use three fully-connected (FC) layers as the classifier, we only use a single fully-connected layer as the classifier. The experimental results show that although there is only a single full connection layer in the network, the classification performance of the network is not much different from that of three full connection layers, but the number of parameters is greatly reduced.

In addition, the Global Average Pooling (GAP) method proposed by Carrer et al. [20] is used in MLES-Net. Gap reduces the number of parameters. On the one hand, it can avoid over fitting, on the other hand, it is more in line with CNN’s working structure: each feature map is associated with the category output, rather than the unit of the feature map is directly associated with the category output. Moreover, GAP considers all local regions to prevent interference by one or two very special regions.

## Implementation details

### Classifiers

In order to study the amount of model calculations and parameters, we compared the parameters and calculations of models by using different classifiers, and different depth models. When using different classifiers to classify the three X-Ray images, the output size of feature map of the last layer of the network is $G \times H \times W$.

GAP: When using a point convolutional layer and GAP being as the classifier, the classifier “GAP” parameter quantity is ${\text{W}} \times 3 + G \times H \times 3 + 3$.

FC: When using the 1-layer “FC” as the classifier, the number of classifier parameters are $3 \times G \times H \times W + 3$.

GAPFC: When a global average pooling layer and a FC layer are used as classifiers, since the pooling layer has no parameters, the parameter of the classifier “GAPFC” is $W + W \times 3 + 3$.

### Parameter comparison

**Fig. 4:**
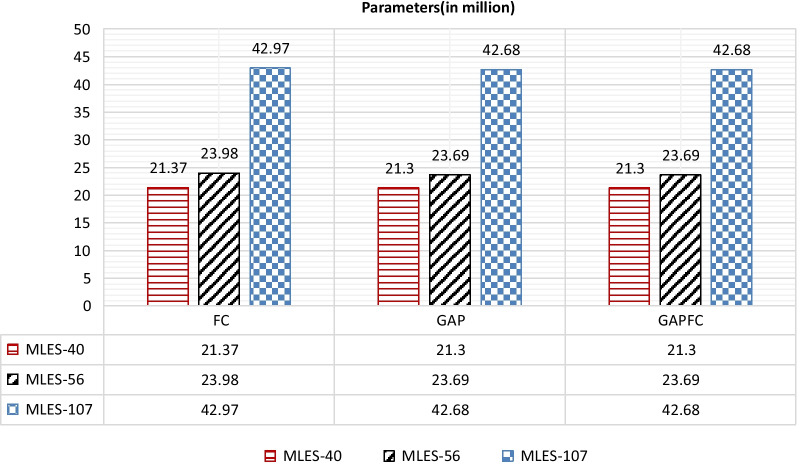
The comparison of parameters of different classifier of MLES-Nets

**Fig. 5:**
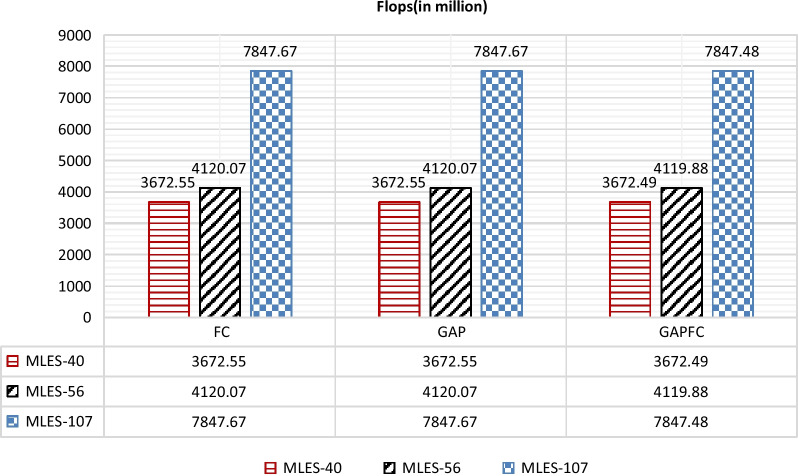
The comparison of FLOPS of different classifier of MLES-Nets

The parameters of different depth networks are shown in Fig. 4, and the amount of calculation is shown in Fig. 5. The numbers of the layers in the network are 40, 56 and 107. The classifiers of the network are FC, GAP and GAPFC.

It can be seen from Fig. 4 that when the network depth is more than 56 layers, the network using FC as the classifier has about 300,000 more parameters than the network using other classifiers. Therefore, we should avoid using FC as a classifier while ensuring accuracy, which can reduce the number of parameters. Although the network parameters of different classifiers are different, the difference is relatively small compared with the total parameters. On the other hand, the parameter quantity of MLES-Net107-FC is 2.0 times that of MLES-Net40-FC and 1.8 times that of MLES-Net56-FC. Therefore, the depth of the network has a greater impact on the parameters of the network model than the classifier. MLES-Net56 is a better choice when the memory of the device is insufficient and the hardware condition does not support too many parameters.

### Calculation comparison

It can be seen from Fig. 5 that the calculation amount is greatly affected by the network depth. The calculation amount of MLES-Net107 is 1.90 times that of MLES-Net56, and 2.14 times that of MLES-Net40. Compared with Net40 and Net56, MELS-Net107 has almost doubled the calculation amount. Therefore, when the model accuracy difference is not large, the MLES-Net56 model has the highest cost performance.

In addition, by comparing the parameter amount and calculation amount of the network using three different classifiers, the network parameter amount using GAP and GAPFC saves about 300,000 parameters than the network using FC. In terms of computation, the network using GAPFC as classifier reduces about 200,000 computation amount compared with the network using GAP and FC as classifier. Therefore, on the premise that accuracy is guaranteed, the network can give priority to the use of GAPFC classifier.

### Data pre-processing

In the experiments, the images in the training set and test are down sampled to a fixed resolution of 224 × 224, and then expand the training set data: Randomly rotate the image between − 10° and 10° with a probability of 0.75, randomly zoom in pictures between 1 and 1.1 times with a probability of 0.75, randomly adjust the brightness of the picture between 0.4 and 0.6 with a probability of 0.75, randomly adjust the random contrast of the picture between 0.8 and 1.25 with a probability of 0.75, and tilt the picture between − 0.2 and 0.2 with a probability of 0.75. After these operations, our training set has been expanded by 4 times, which solves the problem of insufficient X-Ray images to a certain extent and reduces the risk of network overfitting. Our experiments are conducted on the same platform and environment to ensure the credibility of comparisons between different network models. Table 2 shows the software and hardware configuration information of the experimental platform. The “batch size” size of the training set and test set are both set to 12.

**Table 2:** Experimental platform configuration

Attributes	Configuration information
Operating system	Ubuntu 14.04.5 LTS
CPU	Intel(R) Xeon(R) CPU E5-2670 v3 @ 2.30 GHz
GPU	GeForce GTX TITAN X
CUDNN	CUDNN 6.0.21
CUDA	CUDA 8.0.61
Frame	Fastai
IDE	PyCharm
Language	Python

Our training uses the migration learning method. First, we perform pre-training on the ImageNet dataset, and the obtained model parameters are used as the pre-training parameters of our model. We also use the method of pre-training method, that is, gradually increase the learning rate from a small learning rate, and then decay the learning rate. Through repeated experiments, our final training strategy is: The initial learning rate is set to 0.00000005. In 0–40 epochs, the learning rate gradually increases from 0.000004 to 0.0002. In 40–100 epochs, the learning rate is gradually reduced from 0.0002 to 0.000087. A total of 100 epochs are trained.

## Evaluation criteria

In the experiments, we use Accuracy, Precision, Recall, F1-Measure and Specificity as performance indicators. Their formula is as follows:

4 $$Accuracy = \frac{{{\text{TP}} + {\text{TN}}}}{{{\text{TP}} + {\text{TN}} + {\text{FP}} + {\text{FN}}}}$$

5 $$\Pr ecision = \frac{TP}{{TP + FP}},$$

6 $$Recall = \frac{{{\text{TP}}}}{{{\text{TP}} + {\text{FN}}}},$$

7 $$F1 - Measure = 2 * \frac{{{\text{Recall}} * {\text{Precision}}}}{{{\text{Recall}} + {\text{Precision}}}},$$

8 $$Specificity = \frac{{{\text{TN}}}}{{{\text{TN}} + {\text{FP}}}},$$

where, TP is true positive, FP is false positive, FN is false negative, and TN is true negative.

## Results

### Multi-classification image results

In order to verify the effectiveness of MLES module in the model, we introduced the Ablation experiment and used MLES-NET56-GAPFC as the verification model. Under the condition that the number of network layers, classifiers, data sets, experimental platforms and other factors remain unchanged, the MLES module in MLES-NET56-GAPFC is replaced with SE module and SK module, and compared with the original model. The experimental results are shown in Table 3. The results of Table 3 show that the MLES module has effect on the performance improvement of the model. In order to study the influence of depth and classifier on recognition performance, nine kinds of MLES-Nets are used for experiments. The experimental results are shown in Table 4.

**Table 3:** Results of Ablation experiment (%)

Model	Accuracy	Precision	Specificity	F1-Measure
MLES-Net56-GAPFC (without MLES&SE&SK)	91.05	93.57	91.08	92.31
MLES-Net56-GAPFC (SE)	92.01	95.20	91.17	93.14
MLES-Net56-GAPFC (SK)	89.25	92.24	88.11	90.13
MLES-Net56-GAPFC	95.27	96.91	94.66	95.77

**Table 4:** Performance of different depth in MLES-Nets (%)

	Accuracy	Precision	Recall	Specificity	F1-Measure	COVID-19-Acc
*MLES-40*						
FC GAP GAPFC	92.01 91.05 92.98	95.07 92.03 95.40	91.78 90.50 92.65	94.39 94.28 94.40	93.40 91.26 94.00	99.02 95.10 98.00
*MLES-56*						
FC GAP GAPFC	92.56 91.46 **95.27**	95.59 94.58 **96.91**	92.11 91.14 **94.66**	94.74 94.07 **96.49**	93.82 92.82 **95.77**	98.00 98.00 **100.00**
*MLES-107*						
FC GAP GAPFC	92.70 91.73 93.39	94.98 95.05 96.12	92.42 91.44 93.28	95.01 94.28 95.30	93.68 93.21 94.67	98.00 99.02 100.00

It can be seen from Table 4 that the performance of the MLES-Net model using GAP as the classifier is significantly worse than the network model using the other two classifiers, and the performance of the model using GAPFC classifier is better. In Table 4, the significance of bold is to highlight the best comprehensive performance of MLES-Nets with different classifiers and network layers. The performance of MLES-Net56 using the GAPFC classifier (MLES-Net56-GAPFC) is the best, with an accuracy rate of 95.27. Its detection accuracy of COVID-19 is the same as that of MLES-Net107-GAPFC, reaching 100%. Other indicators of MLES-Net56-GAPFC network are also the best, such as precision rate, recall rate, special efficiency and F1-measure, which are 96.91, 94.66, 96.49 and 95.77% respectively. The experimental results show that the performance of the network model does not improve with the increase of the number of network layers.

MLES-Net56-GAPFC and MLES-Net107-GAPFC have the best recognition rate in COVID-19 detection (100%), and the total accuracies rate are also in the front rank, reaching 95.27% and 93.39%, respectively.

### Parameter and calculation

This indicates that as the network deepening, the performance of the network may not continue improving. However, the calculation of MLES-Net107 is 1.90 times of MLES-Net56 and 2.14 times of MLES-Net40. The parameter quantity of MLES-Net107-FC is 1.8 times of MLES-Net56-FC and 2.0 times of MLES-Net40-FC.

### MLES-Net56-GAPFC

**Fig. 6:**
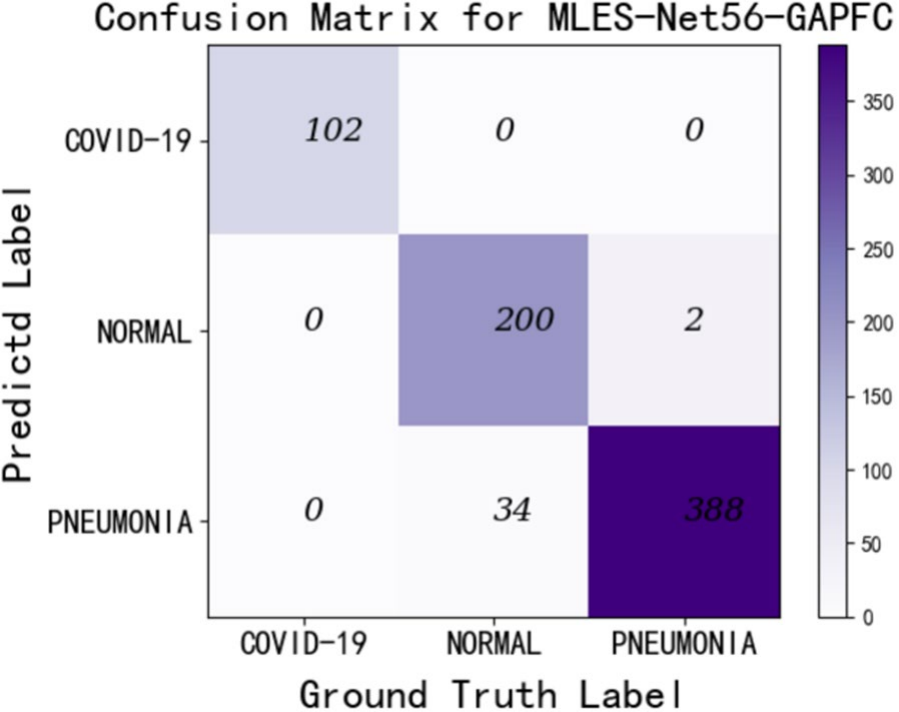
The confusion matrixes of the MLES-Net56-GAPFC

Furthermore, the accuracy rate of MLES-Net56-GAPFC and the recognition rate for COVID-19 reached 95.27% (the highest) and 100%, respectively. Therefore, after comprehensive consideration, we choose MLES-Net56-GAPFC as the first suggested model. In Fig. 6, we give the confusion matrixes of MLES-Net56-GAPFC, and Table 5 gives more detailed results of the recognition performance of MLES-Net56-GAPFC. Figure 6 is the 3-class classification results. The experimental results show that the model has good X-Ray recognition accuracy in patients with COVID-19 and common pneumonia, but a few pictures of normal people's lungs were identified as common pneumonia.

**Table 5:** Accuracy, sensitivity, specificity of MLES-Net56-GAPFC (%)

Class	Accuracy	Sensitivity	Specificity
COVID-19	100	100	100
Pneumonia	99.49	99.49	89.88
Normal	85.47	85.47	99.59
Average	94.99	94.99	96.49

It can be seen from Table 5 that MLES-Net56-GAPFC has excellent performance in X-Ray image recognition for COVID-19-positive patients and patients with common pneumonia. Especially for COVID-19 positive patients, Accuracy, Sensitivity and Sensitivity have reached 100%. However, the accuracy of X-Ray image recognition for normal people is low.

### Comparison with other networks

Next, we further compare the experimental results of MLES-Net56-GAPFC with traditional convolutional networks, such as ResNet-50 [30], DenseNet-121 [8], GoogleNet [26] and VGG-19 [21]. The comparison results are shown in Table 6.

**Table 6:** Performance of other CNNs (%)

Model	Accuracy	Precision	Sensitivity	Specificity	F1-score	COVID-19 Acc
ResNet-50 [14]	93.53	96.01	93.15	96.53	94.56	98.40
DenseNet-121[8]	93.11	95.98	92.75	96.38	94.34	99.02
Google-Net [10]	92.56	95.29	91.56	95.78	93.37	95.10
VggNet-19 [5]	93.11	96.09	92.93	96.47	94.49	100.00
MLES-Net56-GAPFC	95.27	96.91	94.66	96.49	95.77	100.00

ResNet-50 alleviates the problem of gradient explosion and gradient disappearance caused by the increase of depth of the network model through short skip connections. Its recognition rate of COVID-19 is 98.40%, and other performance indicators are better than other network models.

ResNet-50 has reached 98.40% in the recognition rate of COVID-19, and other performance indicators of which are better than other network models for comparing. Although DenseNet-121 and VGG-19 have almost the same recognition rates on our data set, both reach 93.11%. However, because DenseNet-121 uses densely connected blocks, VGG-19 uses three fully-connected layers as a connector, so both of them generate a huge amount of parameters and calculations. GoogleNet has the lowest recognition rate on our data set due to its shallow depth. Because the MLES module enhances the model sensation and semantic region feature learning ability through multi channels, and the MLES-Net56-GAPFC proposed based on this module has no obvious increase in the amount of parameters and calculation, so it is the best among these networks.

After comparing the recognition effect of our model with those of traditional networks on COVID-19 detection, we compare it with the new network models specially designed for the detection of COVID-19. The results are shown in Table 7.

**Table 7:** Comparison of the proposed method with other existing deep learning methods (%)

Name	Class	Method	Accuracy	COVID-19 Acc
Ozturk T [32]	3	Dark-COVID-Net	87.02	98.08
Rajaraman [33]	3	Iteratively pruned deep learning	95.63	99.01
MA Elaziz [34]	3	FrMEMs	96.09	95.09
Mahmud T [35]	3	CovXNet	93.93	96.90
Proposed method	3	MLES-Net56-GAPFC	95.27	100

Although the overall accuracy of MA Elaziz is up to 96.09%, which is 0.82% higher than that of MLES-Net56-GAPFC, its classification accuracy for the COVID-19 detection is only 95.09%, which is 4.91% lower than that of MLES-Net56-GAPFC, and its recognition accuracy rate for COVID-19 detection is the lowest among the networks. Although Dark-COVID-Net has a classification accuracy rate of 98.08% in the COVID-19 detection, its overall accuracy rate is only 87.02%. Combining Tables 4 and 7, the overall accuracy of MLES-Net56-GAPFC's X-Ray image recognition rate and the recognition accuracy for the COVID-19 are very high, both of which indicate that our network’s performance is better and the identification of COVID-19 is more effective.

## Discussion

### Image classification problem

According to the experimental results, both of MLES-NET56-GAPFC and MLES-Net107-GAPFC have a recognition rate of 100% for COVID-19, and the accuracy rate of MLES-Net56-GAPFC is as high as 95.27%. After comprehensive comparison, we choose the MLES-Net56-GAPFC network to compare with other methods. Their best accuracy and COVID-19 category accuracy are 96.09% and 95.09%, respectively, and the overall performance of our model is better than other methods.

By analyzing experimental results, we believe that the convolutional neural network for X-Ray image recognition of COVID-19 should comply with the following principles:

1. The depth of the network model should not be too deep or too shallow. Complex and deep networks such as ResNet have a relatively large amount of parameters and calculations. However, lightweight networks, such as MobileNet, often result in low recognition rates due to insufficient network depth, which hard to get a satisfactory effect.
2. The generalization ability is very important for a network. In the early stage of new coronary pneumonia, chest X-Ray images may have small patchy shadows or interstitial changes, the edge density is shallow and fuzzy, and there is no consolidation feature. In addition, the chest X-Ray images have high degree of similarity between categories and low intra-category variability, which will lead to model deviation and overfitting, resulting in reduced performance and generalization. The MLES module divides the channel into sub-channels with different semantics to enhance the generation of corresponding features in each channel. For ensuring that the model will not be disturbed by one or two very special regions, the model associates the global region with each local region.

The MLES contains Conv1, Conv3, and GAPFC, which reduces the amount of parameters and calculations, and enhances the nonlinear learning ability of the model. Skip connection can increase the network depth, prevent degradation of network.

Meanwhile, through the contrast experiment, we find that with the deepening of network layers, network performance may not continue to improve. In general, MLES-Net56-GAPFC network has the best effect.

We had conducted Ablation experiments for ResNet during the experiment, including feature subsampling using inception model and increasing or decreasing depth or width of ResNet model, but the results were not satisfactory, so it was not included in the experimental results. In this paper, the depth and width of the optimal network model we finally chose were also obtained by comparing the experimental results.

### Other similar studies

At present, the epidemic situation is still spreading all over the world. To some extent, the network proposed in this article can assist medical staff diagnose COVID-19 disease and prevent the continued spread of the epidemic [40]. In the period of global epidemic outbreak, scholars from all over the world have carried out in-depth research on it and achieved good results. Islam et al. [39] introduced the LSTM technology and combined with the deep feature extraction technology of CNN to achieve a high accuracy. Compared with the network model proposed in this paper, their overall accuracy is slightly lower. Saha et al. [41] proposed an automated detection scheme named EMCNet to identify COVID-19 patients by evaluating chest X-Ray images. According to the extracted features, the authors innovatively developed a binary classifier for detecting COVID-19. Through the experimental results, the overall accuracy of EMCNet is slightly higher than that of MLES-Net, but MLES-Net has a higher detection recognition rate for COVID-19 than EMCNet. Islam et al. [42] proposed a combined architecture of CNN and RNN for detecting COVID-19 immunity from chest X-Rays. Although the recognition accuracy of VGG19-RNN is better than that of MLES-Net, the average time per epoch is longer than that of MLES-Net. For machine learning needs a lot of data in the initial iterative learning, the network model may not achieve ideal diagnosis effect in the early stage of epidemic outbreak due to the data lack.

## Conclusions

In this paper, we designed a Multi-level enhance sensation network (MLES-Net) based on the characteristics of COVID-19 X-Ray images. The network has good fitting and generalization ability for the characteristics of high similarity between categories of COVID-19 X-Ray images and low intra-category variability. We used two data sets in the experiments, and find that the MLES-Net can help medical staff diagnose and detect COVID-19 efficiently and stably. Considering the factors such as accuracy rate, number of network model parameters and calculation amount, we believe that the MLES-Net56-GAPFC network model has better practicability. Therefore, the network can provide better help for doctors in detecting COVID-19.

## Data Availability

The datasets used during the current study are available at the website. https://github.com/ieee8023/covid-chestxray-datasethttps://www.kaggle.com/paultimothymooney/chest-xray-pneumonia

## Abbreviations

CNN: Convolutional neural network
MLES: Multi-Level Enhanced Sensation
GAP: Global average pooling layer
FC: Fully connected layer
GAPFC: Global average pooling layer and fully connected layer
RT-PCR: Reverse transcription-polymerase chain reaction
COVID-19: Corona Virus Disease 2019
AI: Artificial intelligence
CCTV: Closed-Circuit Television
CT: Computer tomography
CFWE: Channel feature weight extraction
NN: Neural network
